# Novel *WFS1* variants are associated with different diabetes phenotypes

**DOI:** 10.3389/fgene.2024.1433060

**Published:** 2024-08-16

**Authors:** Lei Wu, Juan Zhang, Danjie Li, Zhongyun Zhang, Qicheng Ni, Rulai Han, Lei Ye, Yifei Zhang, Jie Hong, Weiqing Wang, Guang Ning, Weiqiong Gu

**Affiliations:** ^1^ Shanghai National Clinical Research Center for Metabolic Diseases, Key Laboratory for Endocrine and Metabolic Diseases of the National Health Commission of the PR China, Shanghai National Center for Translational Medicine, Shanghai, China; ^2^ Department of Endocrine and Metabolic Diseases, Shanghai Institute of Endocrine and Metabolic Diseases, Ruijin Hospital, Shanghai Jiao Tong University School of Medicine, Shanghai, China

**Keywords:** WFS1 gene, Wolfram syndrome-like disorders (WSLD), ACMG classification, functional analysis, whole-exome sequencing (WES)

## Abstract

**Background:**

The *WFS1* gene encodes the protein wolframin, which is crucial for maintaining endoplasmic reticulum homeostasis. Variants in this gene are predominantly associated with Wolfram syndrome and have been implicated in other disorders such as diabetes mellitus and psychiatric diseases, which increases the rate of clinical misdiagnosis.

**Methods:**

Patients were diagnosed with early-onset unclassified diabetes according to their clinical and laboratory data. We performed whole-exome sequencing (WES) in 165 patients, interpreting variants according to the American College of Medical Genetics/Association for Molecular Pathology (ACMG/AMP) 2015 guidelines. Variant verification was done by Sanger sequencing. *In vitro* experiments were conducted to evaluate the effects of *WFS1* compound heterozygous variants.

**Results:**

We identified *WFS1* compound heterozygous variants (p.A214fs*74/p.F329I and p.I427S/p.I304T) in two patients with Wolfram Syndrome-Like disorders (WSLD). Both *WFS1* compound heterozygous variants were associated with increased ER stress, reduced cell viability, and decreased SERCA2b mRNA levels. Additionally, pathogenic or likely pathogenic *WFS1* heterozygous variants were identified in the other three patients.

**Conclusion:**

Our results underscore the importance of early genetic testing for diagnosing young-onset diabetes and highlight the clinical relevance of *WFS1* variants in increasing ER stress and reducing cell viability. Incorporating these genetic insights into clinical practice can reduce misdiagnoses and improve treatment strategies for related disorders.

## Introduction

Wolfram syndrome (WS) is a rare autosomal recessive disorder, caused by variants in *WFS1*, which encodes the wolframin protein (Rigoli et al., 2011). The primary phenotypes of WS are diabetes insipidus (DI), diabetes mellitus (DM), optic atrophy (OA), and deafness (D), also known as DIDMOAD syndrome (Barrett et al., 1995). Over 98% of these patients develop diabetes, which is often the first symptom in nearly 80% of cases (Lusk et al., 2020). The clinical diagnosis of WS typically requires the coexistence of diabetes and bilateral optic atrophy (Cano et al., 2007). However, only about half of the cases have the complete DIDMOAD phenotype (de Heredia et al., 2013). Some reports are suggesting that *WFS1* can also cause diabetes in an autosomal dominant mode (Bonnycastle et al., 2013), that *WFS1* heterozygous variants can also cause WS (Gong et al., 2021; Morikawa et al., 2017), and that *WFS1* compound heterozygous variants can also cause diabetes only (Li M. et al., 2020), complicating genotype-phenotype correlations and risking misdiagnosis. Therefore, these heterozygous variants often present with a diverse range of clinical manifestations, from traditional WS symptoms to subtler diabetic features, complicating their diagnosis and subsequent management.

The *WFS1* gene is located on chromosome 4p16.1 and spans a length of 33.4 kb, encoding wolframin protein comprising of 890 amino acids. It consists 8 exons, of which exon 1 is non-coding, and exons 2 to 7 are responsible for encoding the protein, with exon 8 mainly encoding the transmembrane region and the carboxyl terminus (Li L. et al., 2020). Islet β-cells are the primary expression site for *WFS1* (Morikawa et al., 2017). Wolframin is a resident protein in the endoplasmic reticulum (ER) that maintains Ca^2+^ homeostasis and regulates ER stress (Fonseca et al., 2010). The development of diabetes in WS is associated with elevated ER stress in β-cells. The ER is responsible for proper protein folding and degradation of misfolded proteins.

In our study of 165 patients with early-onset unclassified diabetes, we identified two individuals with compound heterozygous *WFS1* variants. We conducted *in vitro* experiments to evaluate the functional impact of compound heterozygous *WFS1* variants. We also found three patients with *WFS1* heterozygous variants considered pathogenic or likely pathogenic (P/LP). This research broadens our understanding of the *WFS1* gene and its phenotypes.

## Materials and methods

### Patients

Between January 2021 and December 2022, we enrolled 225 patients with young-onset diabetes at Ruijin Hospital, affiliated with Shanghai Jiao Tong University School of Medicine. Inclusion criteria were an onset age of ≤30 years and the absence of pancreatic exocrine diseases or other conditions inducing diabetes. Patients were diagnosed using the 2019 WHO guidelines (World Health Organization, 2019). 225 patients were aged 20 [15, 16] and the youngest age of onset was 8 years. All patients were tested for GADA and GADA <7 IU/mL was considered negative. Subsequently, 39 patients with T1DM and 21 with T2DM were excluded. Among the remaining 165 patients with unclassified diabetes, all tested negative for GADA and exhibited no typical T1DM or T2DM phenotypes. Clinical data were collected from the medical records. HbA1c and glucose levels were measured in the central laboratory of Ruijin Hospital using high-performance liquid chromatography and an autoanalyzer, respectively. The study received approval from the Ethics Committee of Ruijin Hospital, and informed consent was obtained from all participants and their relatives.

### Genetic analysis

We used the QIAamp DNA Blood Mini Kit (Qiagen, Germany) to extract genomic DNA according to the manufacturer’s protocol. The integrity and concentration of the extracted DNA were assessed using a NanoDrop spectrophotometer and agarose gel electrophoresis. Library capture was performed using custom probes from Integrated DNA Technologies, Inc. (Integrated DNA Technologies, United States), which were biotinylated to allow for sequence enrichment using the xGenTM Hybridization and Wash Kit (Integrated DNA Technologies, United States). We used the Illumina NextSeq 500 system (Illumina, United States) to sequence captured libraries, and 150 bp paired-end reads were generated, aiming for a minimum average coverage depth of 100x to ensure adequate coverage of exonic regions. We only focused on 52 genes (Supplementary Table S1) and interpreted those genes (Caruso et al., 2014; Hosoe et al., 2017; Guillín-Amarelle et al., 2018; Rutkowska et al., 2022; Bonnefond et al., 2023). Uncommon coding or splicing variants (MAF<1%) in those genes were analyzed according to the ACMG/AMP 2015 guidelines (Richards et al., 2015). All patients and their family members were verified by Sanger sequencing.

### Cell culture

The medium for HEK-293T cells was Dulbecco’s Modified Eagle’s Medium (DMEM) (Meilunbio, China) supplemented with 10% fetal bovine serum (FBS) (Gibco, Canada). HEK-293T cells were cultured at 37°C, under 95% air and 5% CO_2_. We used Lipofectamine 2000 (Invitrogen, United States) to transfect cells with indicated plasmids according to the manufacturer’s instructions.

### Plasmids and luciferase reporter assay

Wild type (WT), p.A214fs*74, p.F329I, p.I427S, p.I304T, p.W690fs*706 and p.E385K were constructed and cloned into pCMV vector (Promega, United States), respectively. Variants of p.W690fs*706 and p.E385K were from two unrelated individuals with WS (Gong et al., 2021; Hu et al., 2022). We used QuickChange Site-Directed Mutagenesis Kit (Stratagene, United States) to generate all *WFS1* variants and Sanger sequencing to verify the full-length coding sequences of all plasmids. HEK-293T cells were seeded in 48-well plates, and 100 ng of ERSE luciferase plasmid and 20 ng of PRL-SV40 plasmid (expressing Renilla luciferase for normalization) were transfected. HEK-293T cells were also co-transfected with 0.2 μg each of WT, p.A214fs*74, p.F329I, p.I427S, p.I304T, p.W690fs*706, p.E385K, and 0.1 μg each of p.A214fs*74, p.F329I, p.I427S, p.I304T. Twenty-4 hours after transfection, cells were stimulated with thapsigargin (TG, ER stress inducer) (10 nM) for 6 h and then harvested. We used the dual-luciferase reporter assay system (Promega, United States) to measure luciferase activity according to the manufacturer’s protocol.

### RNA extraction and real-time PCR

HEK-293T cells were seeded in 48-well plates, transfected with 350 ng of the indicated plasmids (WT, p.A214fs*74, p.F329I, p.I427S, p.I304T, p.W690fs*706, p.E385K) and 175 ng each of p.A214fs*74, p.F329I, p.I427S, p.I304T. RNA was extracted using the EZ-press RNA Purification Kit (EZBioscience, United States) according to the manufacturer’s protocol, and was transcribed into cDNA using the Reverse Transcription System (Promega, United States). An ABI system (Life Technology, United States) and SYBR Green Supermix (Takara, Japan) were used to perform real-time PCR. Primers are shown in the table (Supplementary Table S2).

### Protein extraction and Western blotting

HEK-293T cells were seeded in 24-well plates, transfected with 500 ng each of the indicated plasmids (WT, p.A214fs*74, p.F329I, p.I427S, p.I304T, p.W690fs*706, p.E385K) and 250 ng each of p.A214fs*74, p.F329I, p.I427S, p.I304T. After 48 h, protein samples were isolated with radioimmuno-precipitation assay (RIPA) buffer plus a protease inhibitor cocktail (Thermo Scientific, United States). The primary antibodies were incubated overnight at 4°C and the secondary antibody was incubated for 1 h at room temperature. We used eBlot Touch Imager (eBlot, United States) to visualize bands. The antibodies used are shown in the table (Supplementary Table S3).

### Cell proliferation analysis

HEK-293T cells were seeded in 96-well plates, transfected with 50 ng each of the indicated plasmids (WT, p.A214fs*74, p.F329I, p.I427S, p.I304T, p.W690fs*706, p.E385K) and 25 ng each of p.A214fs*74, p.F329I, p.I427S, p.I304T. After 24 h, we used Cell Counting Kit-8 (CCK-8) (MCE, United States) to measure cell viability. A microplate reader (Bio-Rad, United States) was used to measure OD values at 450 nm wavelength.

### Flow cytometric

HEK-293T cells were seeded in 12-well plates, transfected with 1 μg each of the indicated plasmids (WT, p.A214fs*74, p.F329I, p.I427S, p.I304T, p.W690fs*706, p.E385K) and 500 ng each of p.A214fs*74, p.F329I, p.I427S, p.I304T. We used Annexin V-FITC/PI apoptosis detection kit (YEASEN, China) to stain the cells according to the manufacturer’s protocols. A FACSCalibur™ flow cytometry (BD Biosciences, United States) was used to measure the fluorescent intensity and data were analyzed using FlowJo X 10.0.7 software.

### Bisulfite treatment of DNA and PCR analysis

We used the QIAamp DNA Blood Mini Kit (Qiagen, Germany) to extract genomic DNA according to the manufacturer’s protocol. Isolated genomic DNA was treated with an EZ DNA methylation kit (Zymo Research, United States), and then analyzed by ddPCR. We used ddPCR Supermix for Probes (Bio-Rad, United States) to perform ddPCR, cycling conditions were as follows: 95°C for 10 min, 94°C for 30 s, and 57.5°C for 1 min for 40 cycles. The PCR-amplified 96-well plate was placed into the microtiter analyzer of the QX200 Droplet Reader (Bio-Rad, United States) to detect the fluorescence signals of FAM and VIC (HEX). Then, QuantaSoft Software (Bio-Rad, United States) automatically processed the data to obtain a concentration of methylated and unmethylated *INS* DNA (copies/μL). Then we calculated the ratio of unmethylated to methylated *INS* DNA.

### Statistical analysis

Data are expressed as mean ± SEM. Comparisons between two groups were made by Student’s *t* -test and one-way ANOVA was used to compare three or more groups. Statistics were analyzed by Student’s *t*-test. We used GraphPad Prism V9.0 to analyze all statistics. *P*-value < 0.05 was statistically different.

## Results

**FIGURE 1:**
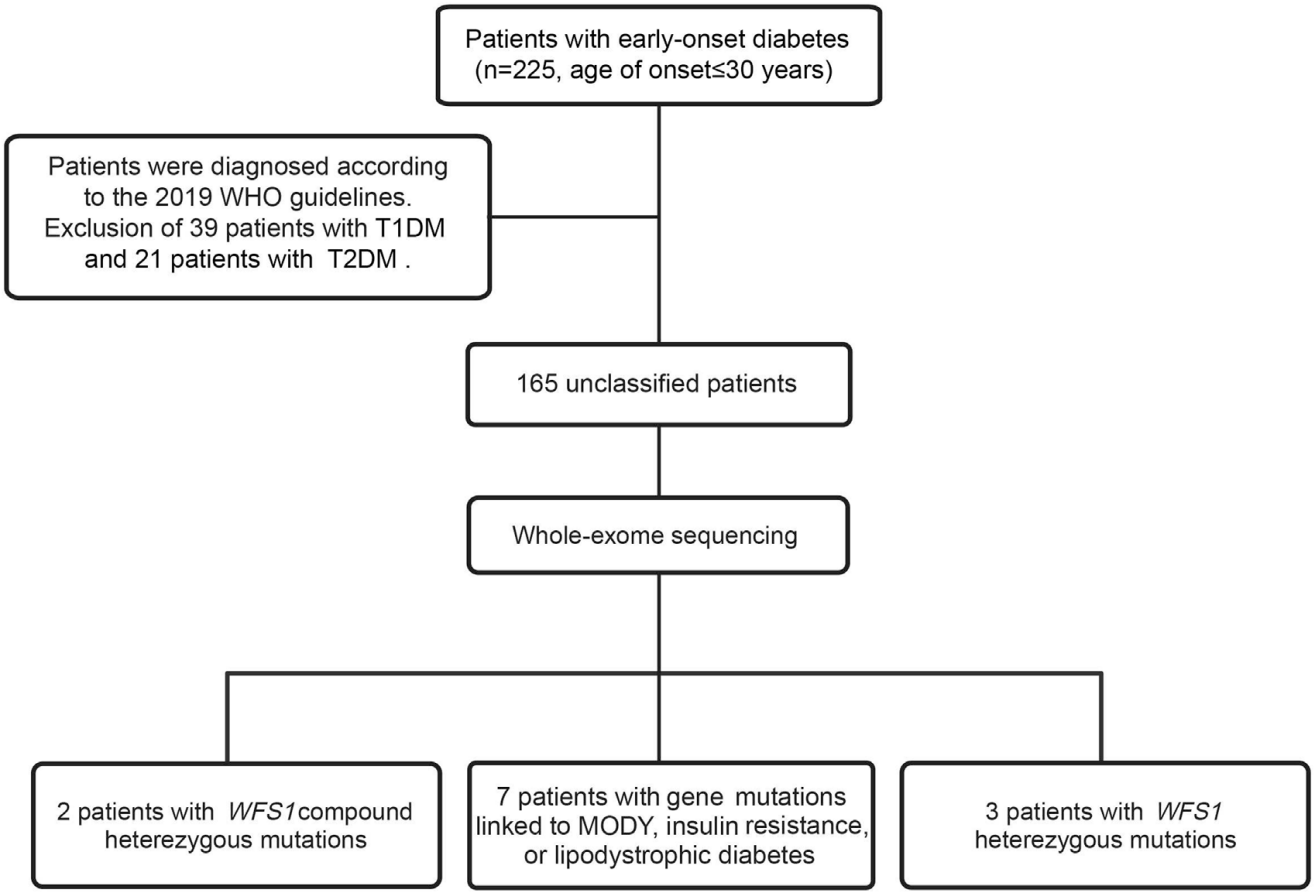
Flowchart of the study.

**FIGURE 2:**
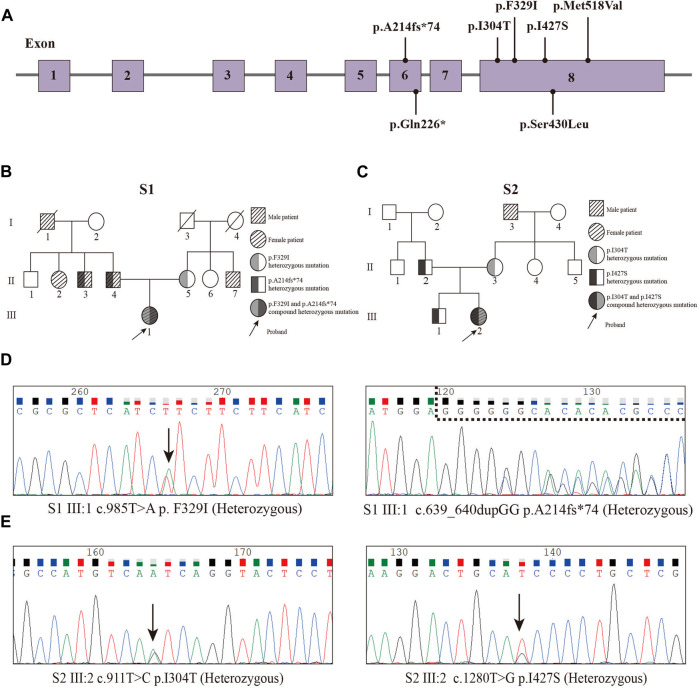
Pedigrees and Sanger sequencing of the two patients (P1 and P2) and locations of variants. **(A)** Locations of variants. **(B)** Pedigree of the S1 family. **(C)** Pedigree of the S2 family. **(D)** Sanger sequencing peak map of the patient 1. **(E)** Sanger sequencing peak map of the patient two.

In our study, we performed WES on 165 patients with young-onset unclassified diabetes (Figure 1). We identified pathogenic/likely pathogenic (P/LP) *WFS1* variants in five patients (Table 1) and other P/LP genes linked to MODY, insulin resistance, or lipoatrophic diabetes in seven patients (Supplementary Table S4). We then focused on *WFS1*. Among these, three patients (2%) exhibited heterozygous variants, while the remaining two patients (1%) had compound heterozygous variants in *WFS1*. Notably, five variants were localized to exon 8, with the remaining two found in exon 6 (Figure 2A). Our analysis classified five variants as P/LP, and two were classified as variants of uncertain significance (VUS) (Table 1).

**TABLE 1:** A summary of *WFS1* compound heterozygous and P/LP *WFS1* heterozygous variants identified in this study.

Patient	Family	Exon	CDS change	AA change	Types of variants	Status	Novel/Reported	Classification
P1	S1	6	c.639_640dupGG	p.A214fs*74	Frameshift	Het	Novel	LP (PVS1, PM2)
		8	c.985T>A	p.F329I	Missense	Het	(Li et al., 2020a)	VUS (PM2, PM3, PP3)
P2	S2	8	c.1280T>G	p.I427S	Missense	Het	(Cano et al., 2007)	P (PS1, PM1, PM2, PP3, PP5)
		8	c.911T>C	p.I304T	Missense	Het	Novel	VUS (PM2, PM3, PP3)
P3	S3	8	c.1289C>T	p.S430L	Missense	Het	(Rohayem et al., 2011)	LP (PM2, PM5, PP3, PP4, PP5)
P4	S4	8	c.1552A>G	p.M518V	Missense	Het	(Matsunaga et al., 2014)	LP (PM1, PM2, PP3, PP4, PP5)
P5	S5	6	c.676C>T	p.Q226*	Nonsense	Het	(Strom et al., 1998)	P (PVS1, PM2, PP4, PP5)

CDS, coding sequence; AA, amino acid. Het, Heterozygous.

PVS, very strong evidence of pathogenicity; PVS1, null variant. PS, strong evidence of pathogenicity; PS1, same amino acid change as previously reported pathogenic variant. PM, moderate evidence of pathogenicity; PM1, mutational hot spot; PM2, variants are absent or at very low frequency in controls; PM3, detected in trans with a pathogenic variant; PM5, same amino acid positions as previously reported pathogenic missense variants. PP, supporting evidence of pathogenicity; PP3, multiple computational predictions suggest that deleterious; PP4, patient’s phenotype or family history is highly correlated with disease; PP5, previously reported as pathogenic; P, pathogenic; LP, likely pathogenic; VUS, variants of uncertain significance.

### Clinical characteristics of patients with *WFS1* compound heterozygous variants

Diagnosed with diabetes at 25, patient one has never experienced ketoacidosis since onset, with GADA negative. Managed effectively with oral medication, she maintains satisfactory HbA1c and C-peptide levels (Table 2). Notably, she exhibits none of the symptoms typically associated with WS, such as DI, OA, or D, and has had no diabetes-related complications to date. Her family history reveals a prominent pattern of diabetes mellitus, with all members developing diabetes at age 35 or older (Figure 2B). Genetic testing showed that this patient carries the *WFS1* compound heterozygous variants (Figure 2D). Sanger sequencing identified heterozygous variants p.A214fs*74 in her father and uncle, and p.F329I in her mother, while her grandmother, aunt, and maternal aunt showed no variants (Supplementary Figure S1). Patient two was first diagnosed with diabetes at 13, presenting with ketoacidosis. At 14, a hospital evaluation indicated average islet function and negative insulin autoantibodies (Table 2). Her clinical profile includes normal urine output and no neurological, psychiatric, or high-frequency hearing issues, or optic nerve atrophy. However, she has a refractive error in her left eye and difficulty distinguishing blue-violet colors. Her medical history includes hydronephrosis, revealed by abdominal ultrasound as bilateral ureteral dilatation, but pancreatic MR scans showed no occupying lesions. Her maternal grandfather, diagnosed with diabetes at age 40, was treated with insulin and medication (Figure 2C). Genetic analysis identified *WFS1* compound heterozygous variants in the patient (Figure 2E). Sanger sequencing in her family revealed heterozygous variants: p.I427S in her father and p.I304T in her mother and brother (Supplementary Figure S2). Other family members were unavailable for genetic testing. According to the guidelines from EURO-WABB (http://euro-wabb.org/guidelines/guidelines/), we can diagnose these patients as WSLD at this moment. Recent findings indicate that certain adolescents exhibiting WSLD present solely with diabetes, leading to frequent misdiagnoses as either T1DM or T2DM (Zalloua et al., 2008; Bonnycastle et al., 2013; Li M. et al., 2020).

**TABLE 2:** Clinical manifestations of patients with P/LP *WFS1* variants.

Patient	P1	P2	P3	P4	P5
Sex (M/F)	F	F	M	F	M
Age of onset (years)	25	13	25	25	25
Age at time of recruitment (years)	30	14	33	25	26
Family history	Yes	Yes	Yes	Yes	Yes
BMI (kg/m^2^)	18.60	20.06	28.20	23.18	22.80
GADA	Neg	Neg	Neg	Neg	Neg
HbA1c (%)	5.6	13.2	11.5	6.4	6.4
FBG (mmol/L)	6.94	11.2	13.27	9.64	7.36
FCP (ng/ml	1.47	1.19	1.42	2.94	2.22
TC (mg/dL)	4.44	4.46	4.93	6.11	5.80
TG (mg/dL)	0.59	0.36	2.04	0.51	3.97
HDL (mg/dL)	1.52	1.76	0.95	2.12	0.90
LDL (mg/dL)	2.74	2.50	2.91	3.82	3.80
DI	—	—	—	—	—
OA	—	—	—	—	—
D	—	—	—	—	—
Renal tract abnormalities	—	Bilateral ureteral dilatation with pelvic separation	—	—	—
Neurological abnormalities	—	—	—	—	—
Current therapy	OAD	Insulin	OAD	OAD	OAD

P/LP, pathogenic/likely pathogenic; F, female; M, male; BMI, body mass index; HbA1c, Hemoglobin A1cOA; FBG, fasting blood glucose; FCP, fasting C-peptide; TC, total cholesterol; TG, triglycerides; HDL, high-density lipoprotein; LDL, low-density lipoprotein; optic atrophy; D, deafness; DI, diabetes insipidus; OAD, oral antidiabetic drugs.

### Functional analysis of *WFS1* compound heterozygous variants

#### Protein expression of wolframin

**FIGURE 3:**
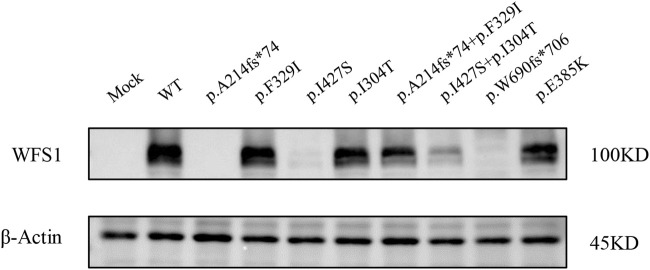
Protein expression levels of Wolframin. (MOCK: transfected with pCMV vector; WT: wild type).

We evaluated wolframin protein expression in HEK cells transfected with *WFS1* variant-carrying plasmids. All four variants, particularly the frameshift variant p.A214fs*74 and the missense variant p.I427S, significantly reduced wolframin levels, with p.A214fs*74 causing complete absence and p.I427S showing near absence of the protein. Combinations of p.A214fs*74 with p.F329I and p.I304T with p.I427S also notably decreased wolframin expression compared to WT (Figure 3). These results suggest that these variants disrupt wolframin protein production, potentially contributing to the development of the associated disease phenotype.

#### Effects on ER stress and UPR

**FIGURE 4:**
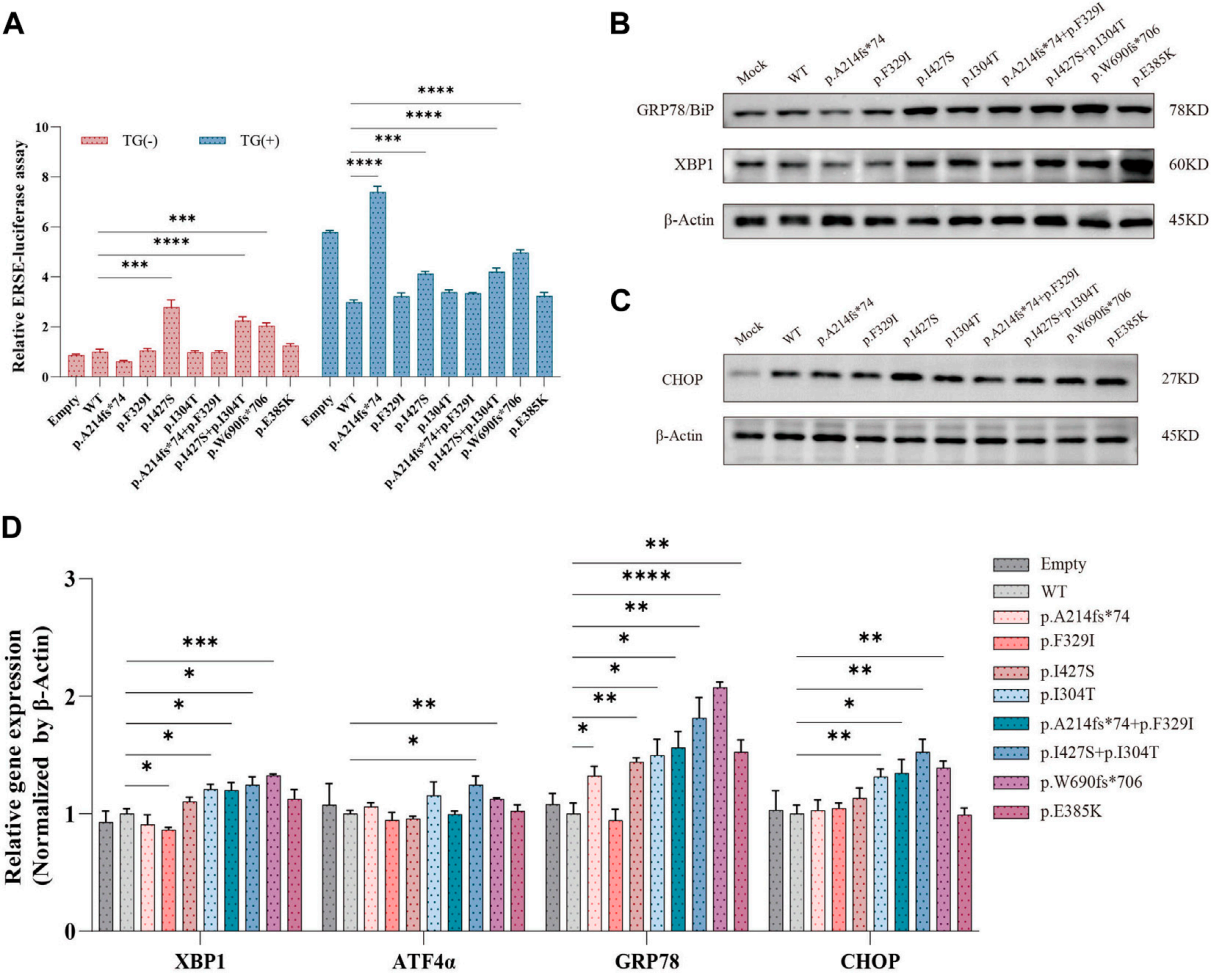
Effects of *WFS1* variants on ER stress and UPR. **(A)** Luciferase reporter assay in HEK-293T cells (n = 4). **(B)** HEK-293T cells transfected with mutant plasmids upregulated ER stress-related molecules. **(C)** Protein expression of CHOP. **(D)** Quantitative PCR for mRNA levels of XBP-1, ATF4α, BiP/GRP78 and CHOP in HEK-293T cells (n = 4). (MOCK/Empty: transfected with pCMV vector; WT: wild type; TG: thapsigargin, ER stress inducer). All statistics were represented as mean ± SEM. *P < 0.05, **P < 0.01 and ***P < 0.001.

Wolframin protein is mainly located in the ER, and variants in the *WFS1* gene cause an imbalance in ER homeostasis, which activates the unfolded protein response (UPR) (Wu and Kaufman, 2006). We performed a dual luciferase reporter gene assay to determine whether *WFS1* variants increase ER stress. The p.I427S and the compound heterozygous p.I427S/p.I304T variants significantly activated the ERSE reporter, both with and without TG stimulation, indicating increased ER stress (Figure 4A). However, the p.I304T variant did not change ERSE activity from the WT. The p.A214fs74 variant significantly increased ER stress enhancer (ERSE) activity under thapsigargin (TG) stimulation, in contrast to the compound heterozygous p.A214fs74/p.F329I and the p.F329I variants (Figure 4A).

Further, protein levels of ER stress markers BiP/GRP78 and XBP-1 were markedly increased in cells with compound heterozygous variants (Figure 4B). Specifically, the p.I427S/p.I304T variant also slightly raised CHOP protein levels (Figure 4C). The single p.I427S and p.I304T variants each upregulated BiP/GRP78 and XBP-1 proteins, with p.I427S additionally boosting CHOP levels (Figures 4B, C). Correspondingly, mRNA expressions of CHOP, BiP/GRP78, and XBP-1 were higher in cells with the compound heterozygous variants (Figure 4D). These results suggest that *WFS1* compound heterozygous variants increase ER stress and activate the UPR pathway.

#### Evaluation of calcium homeostasis and cell apoptosis

**FIGURE 5:**
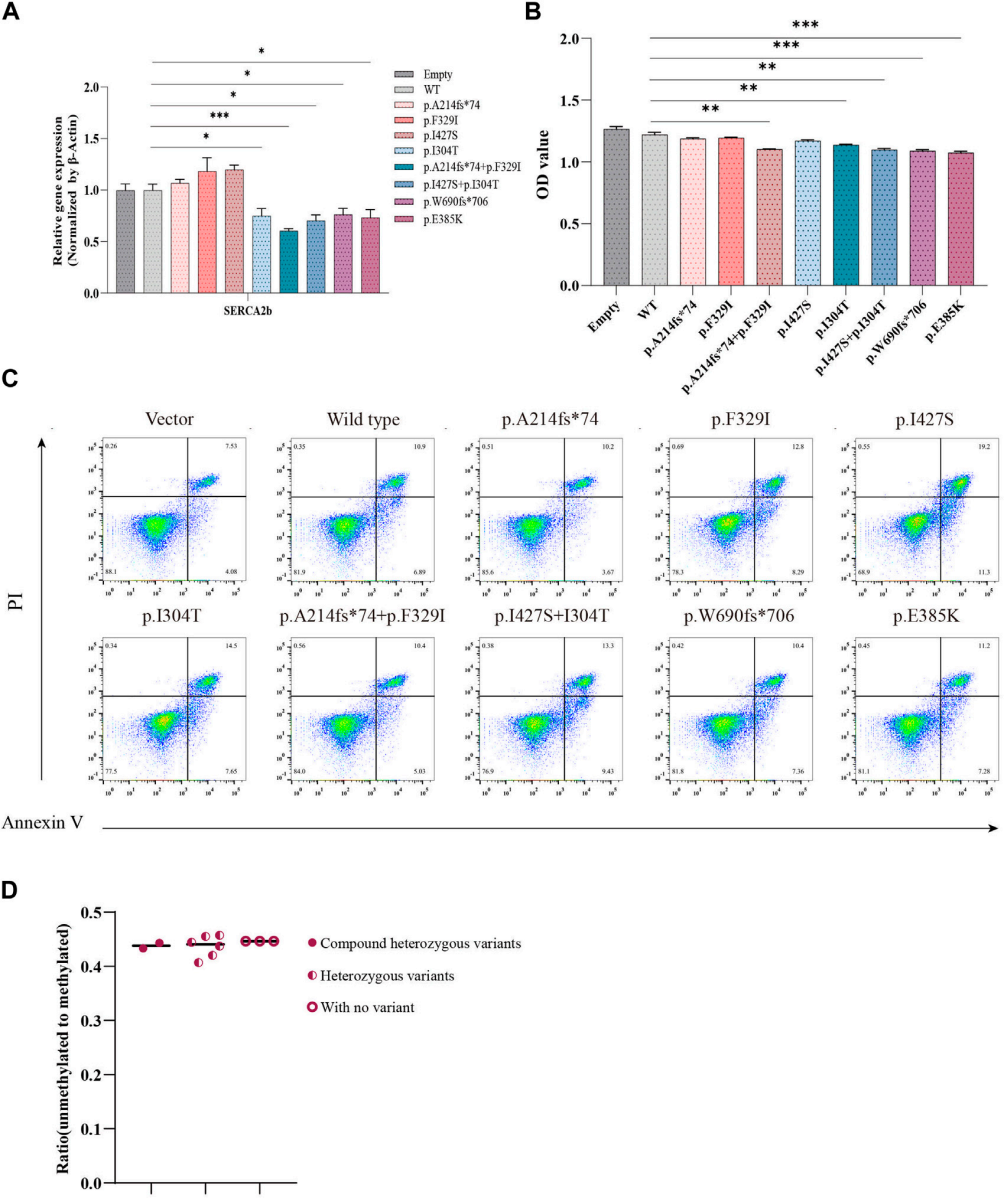
Evaluation of calcium flux and cell proliferation. **(A)** Quantitative PCR analysis of SERCA2b (n = 4). **(B)** Cell proliferation was measured by CCK-8 assay (n = 5). **(C)** Flow cytometry to assess cell apoptosis. **(D)** The extent of pancreatic β cell damage was measured through ddPCR. (Vector/Empty: transfected with pCMV vector. WT: wild type). All data were expressed as mean ± SEM. *P < 0.05, **P < 0.01 and ***P < 0.001.

ER stress is triggered by increased Ca^2+^ efflux from the ER, which leads to an increase in the cytoplasmic Ca^2+^ concentration and consequent activation of calpain-2, resulting in β-cell death (Hara et al., 2014). Wolframin protein is mainly located in the ER and is responsible for maintaining intracellular Ca^2+^ homeostasis by repressing the expression of sarcoendoplasmic reticulum Ca2^+^-ATPase 2b (SERCA2b) (Takei et al., 2006). We examined the impact of *WFS1* compound heterozygous variants (p.A214fs*74/p.F329I and p.I304T/p.I427S) on ER calcium homeostasis. *WFS1* compound heterozygous variants significantly lowered SERCA2b mRNA levels (Figure 5A), disrupting ER calcium balance. Cell viability, measured by CCK-8 assays, was reduced in cells with *WFS1* compound heterozygous variants compared to WT (Figure 5B). Flow cytometry showed no significant increase in apoptosis in cells with *WFS1* compound heterozygous variants compared to WT (Figure 5C). Previous studies have shown that the extent of β-cell death in pancreatic islet cells can be analyzed indirectly using droplet digital PCR (ddPCR). Non-methylated *INS* DNA/methylated *INS* DNA is significantly higher in the diabetic population compared to the normal population (Usmani-Brown et al., 2014). We then analyzed β cell death by ddPCR, it showed no significant increase in β cell death (Figure 5D). This suggests that while *WFS1* compound heterozygous variants impair ER calcium stability and cell viability, they do not significantly heighten β-cell death, providing insights into the cellular effects of *WFS1* variants in diabetes.

### Clinical characteristics of patients with heterozygous *WFS1* variants

In addition to the compound heterozygous variants, we identified three different heterozygous *WFS1* variants in three patients, each classified as P/LP (Table 1). Bonnefond et al. (2023) included the *WFS1* gene in the MODY. Notably, these patients had a significant family history of diabetes, all were diagnosed with diabetes at 25 years old (Supplementary Figure S3; Table 2). They were treated with oral antidiabetic drugs and showed no symptoms of WS. Therefore, these patients were clinically suspected of MODY. The implications of these variants warrant further genetic screening and detailed evaluation of additional family members.

## Discussion

Approximately half of *WFS1* variants are homozygous, but autosomal dominant pathogenic *WFS1* variants have also been identified (Astuti et al., 2017), recessive *WFS1* variants can also lead to syndromic and nonsyndromic diabetes. In patients with WS, homozygous loss-of-function variants are the most common, with fewer missense homozygous variants observed (Bansal et al., 2018). This variation in variant types can lead to clinical misdiagnosis or underdiagnosis due to their varying features.

WS is a rare autosomal recessive neurodegenerative disorder that presents with diabetes insipidus, insulin-deficient diabetes mellitus, optic atrophy, and deafness (Khanim et al., 2001). The most typical manifestation in WS patients is diabetes, but neurological diseases and urinary complications are major contributors to morbidity and mortality in these individuals (Kinsley et al., 1995). Diabetes is commonly diagnosed in the first decade of life, while optic atrophy often becomes evident in the second decade. Urinary and neurological abnormalities generally emerge between the ages of 10 and 30 years (Sherif et al., 2021). Traditionally, the concurrent presence of DM and OA has been considered essential for WS diagnosis, some cases lack early optic atrophy, leading to misdiagnosis as T1DM or T2DM (Zmyslowska et al., 2014; Li et al., 2023). In this research, we detected two patients with compound heterozygous variants in *WFS1* (P1: p.A214fs*74/p.F329I, P2: p.I427S/p.I304T). The p.A214fs*74 and p.I427S variants were classified as LP and P respectively, the other two were classified as VUS, and computational evidences predicted deleterious impacts from these variants. Both patients presented with diabetes mellitus but without optic atrophy. Considering their family history and patient two showed bilateral ureteral dilatation with pelvic separation, it underscored the necessity for comprehensive clinical evaluations in WS and functional analysis to verify the impacts of the two compound heterozygous variants.

Variants in the *WFS1* gene are known to disrupt ER function, linked to β-cell death in permanent neonatal diabetes (Fonseca et al., 2005; Edghill et al., 2008; Bonfanti et al., 2009; Fonseca et al., 2010). The UPR is activated when ER stress occurs, which involves the upregulation of CHOP and transactivation of ATF6α to maintain homeostasis within the ER. As a critical transcription factor, ATF6α activates target genes of the UPR and facilitates the expression of protein-coding genes such as Bip/GRP78, thereby restoring protein folding within the ER lumen (Ye et al., 2000; Wu and Kaufman, 2006). However, *WFS1*, a component of the UPR, negatively regulates the ER stress signaling network by recruiting ATF6α to HRD1, which leads to the degradation of ATF6α and suppression of the UPR. This ultimately leads to a reduction in CHOP, BiP/GRP78, and XBP-1 (Fonseca et al., 2010; Guo et al., 2011). CHOP, a transcriptional repressor, is typically low under homeostatic conditions and is triggered by ER stress (Mv et al., 1994; McCullough et al., 2001; Kwok and Daskal, 2008). Loss-of-function of the *WFS1* gene may result in Ca^2+^ deletion in the ER (Hara et al., 2014), Ca^2+^ homeostasis is essential for maintaining cellular function. SERCA2b is an important gene for maintaining Ca^2+^ balance in the ER, studies have shown that exogenous supplementation of SERCA2b reversed ER Ca^2+^ efflux and prevented cell death (Hara et al., 2014). Thus, SERCA2b downregulation may be detrimental to maintaining ER Ca^2+^ homeostasis. Subsequently, elevated cytosolic Ca^2+^ induced cell death. Our *in vitro* studies showed that protein expression of *WFS1* compound heterozygous variants (p.A214fs*74/p.F329I and p.I304T/p.I427S) significantly decreased compared to WT. The apparent decrease in wolframin protein in the double p.I304T/p.I427S transfection versus the single p.I304T transfection may include a dominant negative effect of the p.I427S variant when co-expressed with p.I304T, potentially through misfolding and subsequent degradation of the protein, or by interference with the translation machinery. *WFS1* compound heterozygous variants (p.A214fs*74/p.F329I and p.I304T/p.I427S) upregulated the UPR pathway and decreased SERCA2b mRNA levels, indicating increased calcium efflux. This led to reduced cell viability, while flow cytometry and *INS* DNA methylation analyses showed no significant differences in apoptosis or β-cell death. Reduced cell viability, in the absence of a noticeable increase in apoptosis or beta-cell death, may be attributed to mitochondrial dysfunction. This dysfunction can diminish cellular viability by impairing ATP production and elevating oxidative stress. Additionally, alterations in the cell cycle can induce a quiescent state that inhibits cell proliferation without leading to cell death. In addition, p.I304T/p.I427S resulted in more severe functional impairment than p.A214fs*74/p.F329I, which may explain why patient two showed a more severe phenotype than patient one.

The pathogenicity of *WFS1* variants correlates with their location in the gene. Variants in the luminal domain often lead to early-onset diabetes and typical WS symptoms, while those in the transmembrane and cytoplasmic domains are associated with milder, later-onset forms (Qian et al., 2015). Patient one, with the p.A214fs*74 variant in the cytoplasmic domain and the p.F329I variant in the transmembrane domain (Supplementary Figure S4), was diagnosed with diabetes at 25 but showed no WS symptoms for 7 years. Patient two, carried the p.I304T variant in the cytoplasmic domain and the p.I427S in the luminal domain (Supplementary Figure S4), which may explain why she had an earlier onset and a heavier phenotype than patient one. One report indicated delayed onset of optic atrophy at age 53 in a patient first diagnosed with diabetes at age 33 (Lieber et al., 2012), suggesting the potential for later emergence of WS symptoms. Another study demonstrated that an individual diagnosed with early-onset diabetes was found to be homozygous for the *WFS1* gene, yet did not exhibit the additional clinical features typically associated with WS (Bansal et al., 2017). Therefore, we verified the two *WFS1* compound heterozygous variants indeed lead to impaired function. We still cannot diagnose them as WS, but now as WSLD. Until now, no studies have shown that isolated diabetes progresses to WS, so this needs to be followed up closely with our patients later.

We also identified three patients with P/LP heterozygous variants in *WFS1*. Notably, these patients had a strong family history of diabetes and were managed with oral antidiabetic drugs. We, therefore, suspected them with MODY, follow-up is needed as well as family line validation. The demographic and clinical analysis of the five patients with P/LP *WFS1* variants in our study has uncovered significant patterns that contribute to the understanding of diabetes phenotypes associated with *WFS1* variants. Heterozygous variants in the *WFS1* gene present considerable diagnostic challenges in WS, mainly because their clinical manifestations are often milder and less distinct than those seen in homozygous cases. This variability complicates clinical identification and timely diagnosis of WS, potentially delaying appropriate management and counseling, which could impact patient outcomes. Therefore, a deeper understanding of the impact of these heterozygous variants is essential for refining diagnostic criteria and developing targeted therapeutic strategies.

Wolfram syndrome is also associated with optic atrophy, hearing loss, and neurodegeneration, among other issues. Early genetic diagnosis could facilitate the monitoring of these comorbidities and the initiation of supportive therapies. Several classes of drugs have been reported for the treatment of WS, including dantrolene sodium (Nguyen et al., 2020), 4-phenylbutyric acid (4-PBA) and tauroursodeoxycholic acid (TUDCA) (Kitamura et al., 2022), glucagon-like peptide (GLP)-1 receptor agonists (Sedman et al., 2016; Kondo et al., 2018), and valproic acid (Kakiuchi et al., 2009). We are going to closely follow up on the two patients identified as harboring *WFS1* compound heterozygous variants with targeted examinations as well as targeted medications.

However, the study’s limitations include a small sample size and a lack of ethnic diversity, which may affect the generalizability of the findings. Future studies involving larger and more diverse cohorts are essential to further validate and enhance our understanding of the role of *WFS1* variants in diabetes.

In conclusion, we identified two *WFS1* compound heterozygous variants in patients with WSLD. The functional analysis verified the impaired function of the *WFS1* compound heterozygous variants. The *WFS1* gene is associated with diverse phenotypes, ranging from nonsyndromic diabetes to syndromic conditions. Early genetic screening is crucial for optimal patient management and timely intervention as the disease progresses.

## Data Availability

The original contributions presented in the study are included in the article/Supplementary Material, further inquiries can be directed to the corresponding author.
