# Supplementary material for: Novel WFS1 variants are associated with different diabetes phenotypes
Source: Front Genet. 2024 Aug 16;15:1433060. doi: 10.3389/fgene.2024.1433060 (PMC11361961; doi:10.3389/fgene.2024.1433060)
Supplement: Supplementary file 1 [file Table1.docx]

**Supplementary table 1** Genes were analyzed in the WES.

| **Gene** | **Inheritance mode** | **Genomic location** | |
| --- | --- | --- | --- |
| *GCK* | Autosomal dominant or recessive | 7p13 | MODY (without other atypical features) |
| *HNF1A* | Autosomal dominant | 12q24.31 |  |
| *HNF4A* | Autosomal dominant | 20q13.12 |  |
| *PDX1* | Autosomal dominant | 13q12.2 |  |
| *HNF1B* | Autosomal dominant | 17q12 |  |
| *NEUROD1* | Autosomal dominant | 2q31.3 |  |
| *CEL* | Autosomal dominant | 9q34.13 |  |
| *INS* | Autosomal dominant | 11p15.5 |  |
| *ABCC8* | Autosomal dominant | 11p15.1 |  |
| *KCNJ11* | Autosomal dominant | 11p15.1 |  |
| *APPL1* | Autosomal dominant | 3p14.3 |  |
| *GATA6* | Autosomal dominant | 18q11.2 |  |
| *WFS1* | Autosomal dominant or recessive | 4p16.1 |  |
| *TRMT10A* | Autosomal recessive | 4q23 |  |
| *PCBD1* | Autosomal recessive | 10q22.1 |  |
| *GATA4* | Autosomal dominant | 8p23.1 |  |
| *RFX6* | Autosomal dominant | 6q22.1 |  |
| *MAFA* | Autosomal dominant | 8q24.3 |  |
| *SLC19A2* | Autosomal dominant or recessive | 1q24.2 |  |
| *ONECUT1* | Autosomal dominant | 15q21.3 |  |
| *KCNK16* | Autosomal dominant | 6p21.2 |  |
| *HNF1B* | Autosomal dominant | 17q12 | Neonatal diabetes (without other atypical features) |
| *GCK* | Autosomal dominant or recessive | 7p13 |  |
| *INS* | Autosomal dominant | 11p15.5 |  |
| *ABCC8* | Autosomal dominant | 11p15.1 |  |
| *KCNJ11* | Autosomal dominant | 11p15.1 |  |
| *GATA6* | Autosomal dominant | 18q11.2 |  |
| *GATA4* | Autosomal dominant | 8p23.1 |  |
| *AIRE* | Autosomal dominant or recessive | 21q22.3 | Syndromic, including neonatal and early-onset diabetes  Syndromic, including neonatal and early-onset diabetes |
| *PDX1* | Autosomal dominant | 13q12.2 |  |
| *SLC2A2* | Autosomal recessive | 3q26.2 |  |
| *WFS1* | Autosomal dominant or recessive | 4p16.1 |  |
| *SLC19A2* | Autosomal dominant or recessive | 1q24.2 |  |
| *EIF2AK3* | Autosomal recessive | 2p11.2 |  |
| *FOXP3* | X-linked recessive | Xp11.23 |  |
| *PTF1A* | Autosomal recessive | 10p12.2 |  |
| *GLIS3* | Autosomal recessive | 9p24.2 |  |
| *CEL* | Autosomal dominant | 9q34.13 |  |
| *PAX6* | Autosomal recessive | 11p13 |  |
| *STAT5B* | Autosomal recessive | 17q21.2 |  |
| *CISD2* | Autosomal recessive | 4q24 |  |
| *RFX6* | Autosomal dominant | 6q22.1 |  |
| *NEUROD1* | Autosomal dominant | 2q31.3 |  |
| *NEUROG3* | Autosomal recessive | 10q22.1 |  |
| *IER3IP1* | Autosomal recessive | 18q21.1 |  |
| *ZFP57* | Autosomal recessive | 6p22.1 |  |
| *MNX1* | Autosomal recessive | 7q36.3 |  |
| *GATA6* | Autosomal dominant | 18q11.2 |  |
| *DNAJC3* | Autosomal recessive | 13q32.1 |  |
| *STAT3* | Autosomal dominant | 17q21.2 |  |
| *TRMT10A* | Autosomal recessive | 4q23 |  |
| *NKX2-2* | Autosomal recessive | 20p11.22 |  |
| *CDKN1C* | Autosomal dominant | 11p15.4 |  |
| *PPP1R15B* | Autosomal recessive | 1q32.1 |  |
| *LRBA* | Autosomal recessive | 4q31.3 |  |
| *IL2RA* | Autosomal recessive | 10p15.1 |  |
| *FOXA2* | Autosomal dominant | 20p11.21 |  |
| *CNOT1* | Autosomal dominant | 16q21 |  |
| *YIPF5* | Autosomal recessive | 5q31.3 |  |
| *EIF2B1* | Autosomal dominant | 12q24.31 |  |
| *MIA3* | Autosomal recessive | 1q41 |  |
| *STAT1* | Autosomal dominant | 2q32.2 |  |
| *MANF* | Autosomal recessive | 3p21.2 |  |
| *ONECUT1* | Autosomal dominant | 15q21.3 |  |
| *IRS1* | Autosomal dominant | Xp22.1 | Lipoatrophic Diabetes + Insulin Resistance |
| *PPARG* | Autosomal dominant | 3p25 |  |
| *LMNA* | Autosomal dominant | 1q22 |  |
| *INSR* | Autosomal dominant or recessive | 19P12.2 |  |
