# Supplementary material for: Novel WFS1 variants are associated with different diabetes phenotypes
Source: Front Genet. 2024 Aug 16;15:1433060. doi: 10.3389/fgene.2024.1433060 (PMC11361961; doi:10.3389/fgene.2024.1433060)
Supplement: Supplementary file 2 [file Image4.pdf]

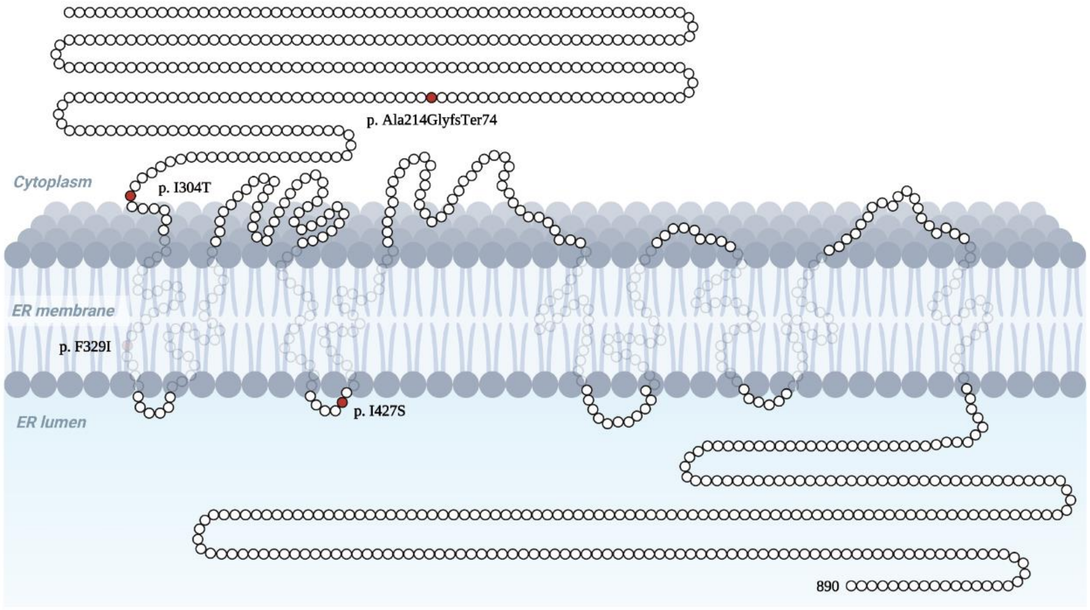

Supplementary Figure 4. Shema of the *WFS1* gene. Locations of the two *WFS1* compound heterozygous variants. Created with BioRender.com.
