# Supplementary material for: Novel WFS1 variants are associated with different diabetes phenotypes
Source: Front Genet. 2024 Aug 16;15:1433060. doi: 10.3389/fgene.2024.1433060 (PMC11361961; doi:10.3389/fgene.2024.1433060)
Supplement: Supplementary file 3 [file Image2.pdf]

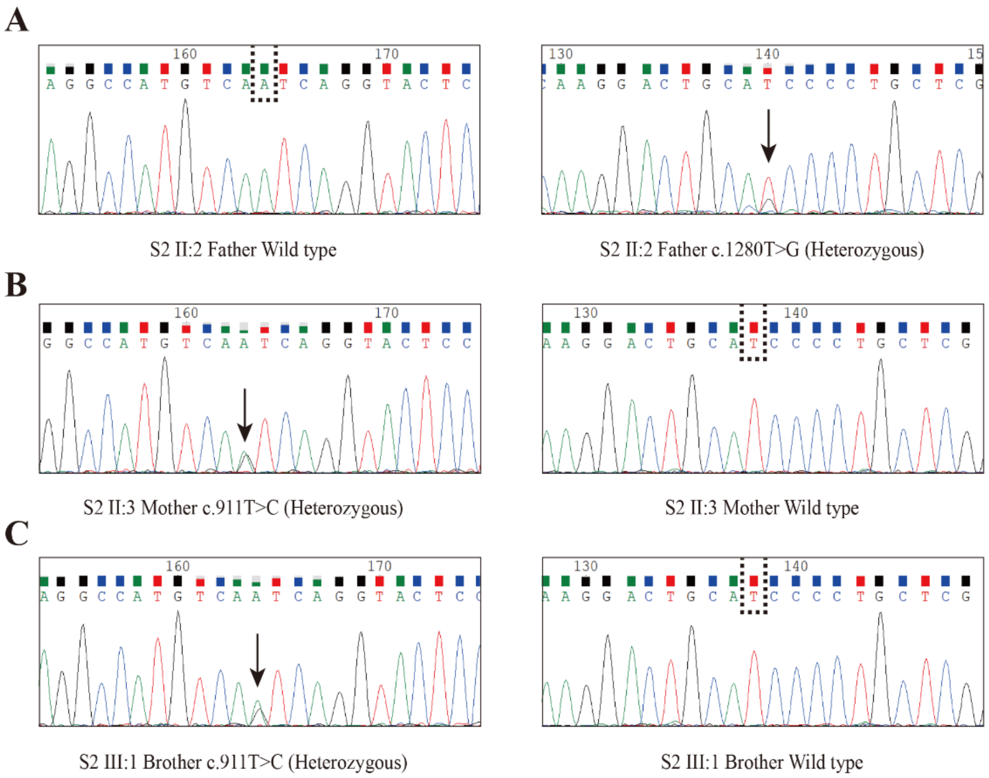

Supplementary Figure 2. PCR sequencing of the S2 family.
Patient 2’s father was heterozygous for c.1280T>G (p.I427S), and her mother and brother were heterozygous for c.911T>C (P.I304T).
