# Supplementary material for: Novel WFS1 variants are associated with different diabetes phenotypes
Source: Front Genet. 2024 Aug 16;15:1433060. doi: 10.3389/fgene.2024.1433060 (PMC11361961; doi:10.3389/fgene.2024.1433060)
Supplement: Supplementary file 4 [file Image3.pdf]

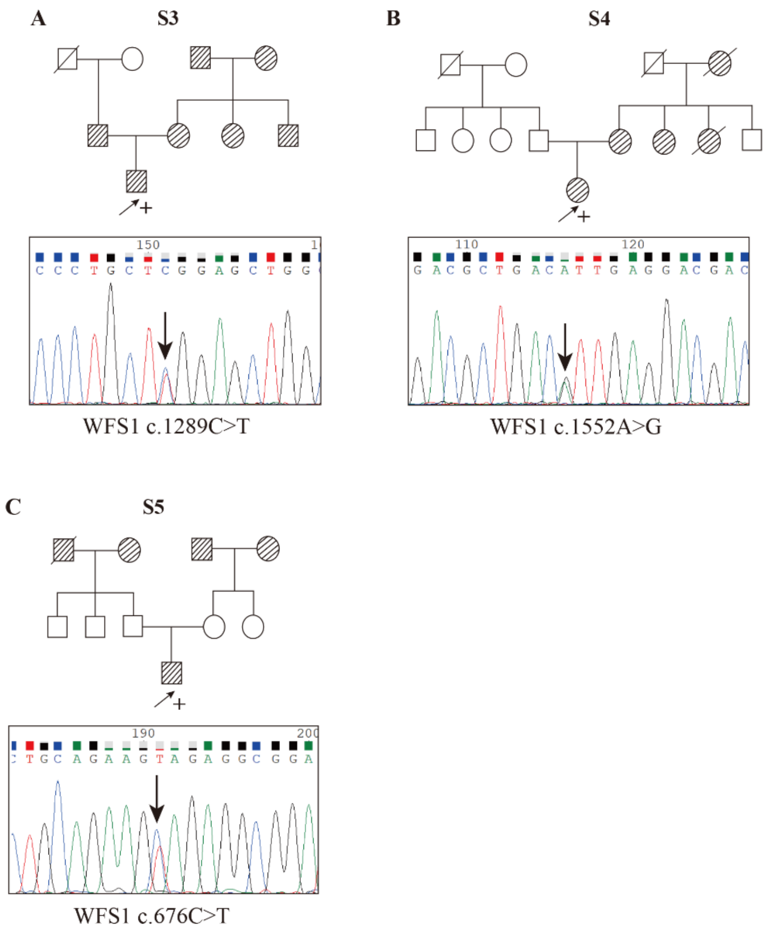

Supplementary Figure 3. Pedigrees and Sanger sequencing of the three (P3, P4 and P5) patients. (A)Pedigrees and Sanger sequencing of the S3 family. (B)Pedigrees and Sanger sequencing of the S4 family. (C)Pedigrees and Sanger sequencing of the S5 family. +: Genetic testing performed; Slash means diabetic.
