# Supplementary material for: Novel WFS1 variants are associated with different diabetes phenotypes
Source: Front Genet. 2024 Aug 16;15:1433060. doi: 10.3389/fgene.2024.1433060 (PMC11361961; doi:10.3389/fgene.2024.1433060)
Supplement: Supplementary file 5 [file Table2.docx]

**Supplementary table 2** Primers used in this study.

| Gene | Forward | Reverse |
| --- | --- | --- |
| human β-actin | 5′-CATGTACGTTGCTATCCAGGC-3′ | 5′CTCCTTAATGTCACGCACGAT-3′ |
| human WFS1 | 5′-AGAACGAACGGGAGGTGA-3′ | 5′-TCTTGGACTCGCTGCTGA-3′ |
| human GRP78/Bip | 5′-CATCACGCCGTCCTATGTCG-3′ | 5′-CGTCAAAGACCGTGTTCTCG-3′ |
| human XBP1 | 5′-GGATTCTGGCGGTATTGA-3′ | 5′-AAAGGGAGGCTGGTAAGG-3′ |
| human ATF4 | 5’-GACCGAAATGAGCTTCCTGA-3’ | 5’-ACCCATGAGGTTTGAAGTGC-3’ |
| human SERCA2b | 5′-CGAACCCTTGCCACTCATCTTC3’ | 5′-TGCCGAGAACGAGCAGGATTTG-3′ |
| human CHOP | 5′-AGAACCAGGAAACGGAA ACAGA-3’ | 5′-TCTCCTTCATGCGCTGCTTT-3’ |
