# Supplementary material for: Novel WFS1 variants are associated with different diabetes phenotypes
Source: Front Genet. 2024 Aug 16;15:1433060. doi: 10.3389/fgene.2024.1433060 (PMC11361961; doi:10.3389/fgene.2024.1433060)
Supplement: Supplementary file 6 [file Table3.docx]

**Supplementary table 3** Antibodies used in this study.

| Antibody | Catalog |
| --- | --- |
| β-Actin (13E5) Rabbit mAb | #5125S (Cell Signaling) |
| WFS1 Antibody | #8749S (Cell Signaling) |
| CHOP(D46F1) Rabbit mAb | #5554S (Cell Signaling) |
| XBP-1s (D2C1F) Rabbit mAb | #12782S (Cell Signaling) |
| BiP (C50B12) Rabbit mAb | #3177S (Cell Signaling) |
