# Supplementary material for: Novel WFS1 variants are associated with different diabetes phenotypes
Source: Front Genet. 2024 Aug 16;15:1433060. doi: 10.3389/fgene.2024.1433060 (PMC11361961; doi:10.3389/fgene.2024.1433060)
Supplement: Supplementary file 7 [file Table4.docx]

**Supplementary table 4** Other genes identified in this study.

| Patient | Gene | CDS change | AA change | Status | Classification | Novel/Reported |
| --- | --- | --- | --- | --- | --- | --- |
| P6 | *LMNA* | c.1745G>A | p.Arg582His | Het | L.P | Reported |
| P7 | *GCK* | c.998C>G | p.Thr333Arg | Het | L.P | Novel |
| P8 | *HNF1A* | c.754C>T | p.Gln252* | Het | P | Novel |
| P9 | *HNF1A* | c.1069G>T | p.Glu357* | Het | P | Novel |
| P10 | *NEUROD1* | c.616_617insC | p.His206Profs*38 | Het | P | Reported |
| P11 | *KCNJ11* | c.685G>A | p.Glu229Lys | Het | P | Reported |
| P12 | *INSR* | c.3164C>T | p.Ala1055Val | Het | P | Reported |

CDS, coding sequence; AA, amino acid; Het, heterozygous; L.P, Likely pathogenic; P, Pathogenic.
