# Supplementary material for: Novel WFS1 variants are associated with different diabetes phenotypes
Source: Front Genet. 2024 Aug 16;15:1433060. doi: 10.3389/fgene.2024.1433060 (PMC11361961; doi:10.3389/fgene.2024.1433060)
Supplement: Supplementary file 8 [file Image1.pdf]

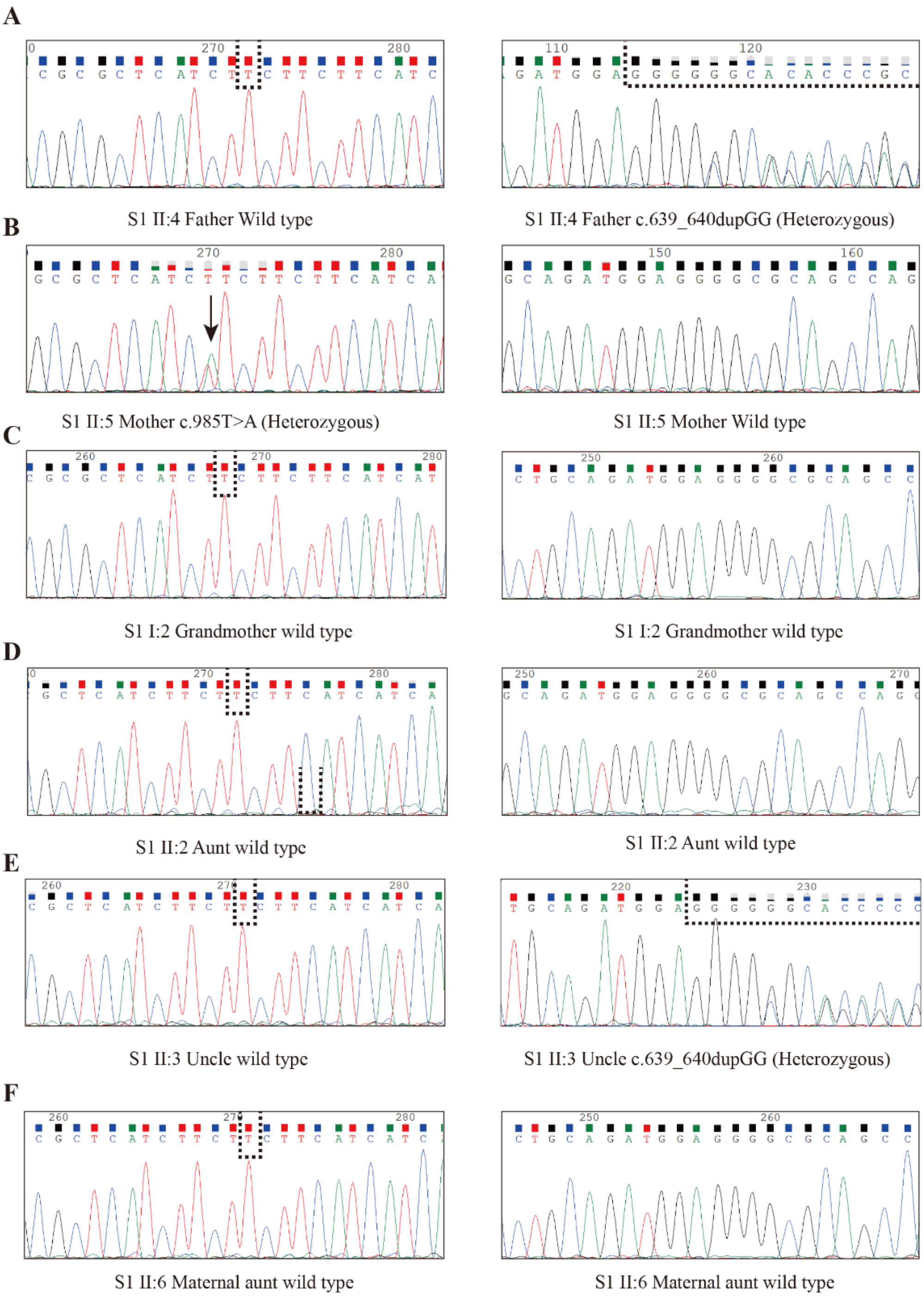

Supplementary Figure 1. PCR sequencing of the S1 family.
Patient 1's father and uncle were heterozygous for c.985T>A (p.F329I), and her mother was heterozygous for c.639_640dupGG (P.A214fs*74).
